# Threshold Effect of Foreign Direct Investment and Carbon Emissions Performance From the Perspective of Marketization Level: Implications for the Green Economy

**DOI:** 10.3389/fpsyg.2021.708749

**Published:** 2021-09-27

**Authors:** Hao Hu, Haiyan Wang, Shuang Zhao, Xun Xi, Lan Li, Xiaojiao Shi, Yingzi Lu, Jianping Yu, Xiaoxiao Liu, Jun Li, Haiyan Zhou

**Affiliations:** ^1^School of Economics, Shanghai University, Shanghai, China; ^2^School of Economics, East China Normal University, Shanghai, China; ^3^School of Management, Jiangsu University, Zhenjiang, China; ^4^Institute of International Business, Zhejiang Gongshang University, Hangzhou, China; ^5^School of Business Administration, Henan University of Economics and Law, Zhengzhou, China; ^6^Research Center of Henan Economy, Henan University of Economics and Law, Zhengzhou, China; ^7^School of Business Administration, Jimei University, Xiamen, China; ^8^School of Business Administration, Wenzhou Polytechnic, Wenzhou, China; ^9^China Center for Economic Research, East China Normal University, Shanghai, China

**Keywords:** foreign direct investment, carbon intensity, marketization level, threshold effect, Yangtze River Economic Belt

## Abstract

Exploring the path and mechanism of marketization level in the effect of foreign direct investment (FDI) on carbon emission performance will help to maximize the stimulation effect of foreign investment on green and low-carbon development. This study used the panel data of 11 provinces and cities in the Yangtze River Economic Belt from 2008 to 2016. A panel threshold model is constructed to explore the non-linear relationship between FDI and carbon emissions performance from the perspective of marketization level. The main conclusions are as follows: First, from the perspective of marketization level, a significant double threshold effect exists between foreign participation and carbon emission intensity, with threshold values of 4.4701 and 9.2516 respectively. Second, as the marketization level increases, the technology spillover effect of FDI increases, and the stimulation effect of foreign participation on carbon intensity decreases significantly, but it does not inhibit carbon intensity, indicating that the overall benefits brought by FDI technology spillovers are still insufficient to offset pollution caused by foreign investment. Third, the eastern region of the Yangtze River Economic Belt has crossed the second threshold. In the central and western regions, the marketization process is relatively slow except for Chongqing, and the regions are still firmly stuck between the first and second thresholds. In response to the conclusions of the empirical research, relevant policy suggestions are put forward from three dimensions, namely, the strategy of introducing foreign investment, construction of the marketization system, and environmental regulation, to achieve low-carbon and green development in the Yangtze River Economic Belt.

## Introduction

Since the 1980s, with the intensification of reform and opening up, a large amount of foreign direct investment (FDI) has flowed into China to implement the strategy of “trading domestic market for technology.” In 2014, China surpassed the United States as the country with the largest FDI inflow, with an investment volume of 129 billion USD. On one hand, the inflow of FDI has alleviated the problem of capital shortage during economic development to a certain extent and promoted employment effectively. At the same time, FDI also has a complex and profound impact on the environment of China. If FDI is a crucial driving force of the green economy, then the level of marketization is an important guarantee for the green economy. In addition, the level of marketization is one of the important factors that determine the intensity of the technology spillover of FDI. There are significant regional development disparities in China, and the levels of marketization in the regions are also uneven. As FDI affects carbon emissions, how do the marketization factors exert their effect in the process? Are there significant differences in the effects of FDI on carbon emissions under different market conditions? Based on the perspective of marketization level, this study selects a typical region, the 11 provinces and cities in the Yangtze River Economic Belt, as the research object. It analyses the mechanism and path of marketization factors in the process of FDI affecting carbon emissions, and the non-linear relationship between FDI and carbon emissions under different marketization levels. It is hoped that the results can be used as academic references for formulating reasonable regional economic policies, deepening the construction of marketization, and implementing low-carbon and green development.

## Literature Review

The core of green economic growth is to realize as much economic growth as possible with as few resources and environmental costs as possible, that is, to enhance the efficiency of green economic growth (Xu et al., 2017). Such enhancement mainly depends on the overall improvement of the country’s level of green technology (Letchumanan and Kodama, 2000; Xu et al., 2017). As a major way to achieve technology progress, the introduction of foreign capital has an uncertain effect on the efficiency of green economic growth. Some researchers believe that the introduction of foreign capital promotes the enhancement of the efficiency of China’s green economic growth. However, some studies have shown that the introduction of foreign capital inhibits the efficiency in the short run. Other studies have concluded that this method of technological progress has different effects on the efficiency of green economic growth. For example, Hermes and Lensink (2003) used China’s provincial panel data and revealed that technological innovation that is measured by R&D expenditure enhances the efficiency of green economic growth, while at the same time, technology import measured by FDI inhibits the efficiency. Wang et al. (2021) specifically highlighted that the introduction of foreign capital has uncertain effects on the efficiency of regional green economic growth. The reasons the method of technological progress has uncertain effects on the enhancement of such efficiency can be explained in two aspects. First, there is uncertainty as to whether the method of progress can result in effective technological progress. For example, technological innovation faces problems such as opportunity cost and constraints in basic research conditions (Girma, 2010; Hu et al., 2020). The introduction of foreign investment and technology face problems such as technology matching, as well as poor digestion and absorption in the region that introduces the technology. On the other hand, even if the introduction of foreign capital can result in effective technological progress, whether it can enhance the efficiency of green economic growth depends on the bias of the technological progress formed. When that formed by the introduction of foreign capital is a type of green technological progress biased toward environmental improvement, it enhances the efficiency of green economic growth. When the situation is reversed, it hinders the enhancement (Alfaro et al., 2010; Hu et al., 2021).

Relatively more studies have examined FDI technology spillovers. MacDougall (1960) was one of the first to explore the effect of such spillovers on the host countries. Many scholars have conducted research and exploration on FDI technology spillovers from various aspects such as industrial linkage, competitive effects, and talent flow, while three major viewpoints have been formed, namely positive, negative, and insignificant. One of the important reasons for the existence of differential effects in host countries is the significant differences in their economic and social environments. FDI technology spillover is a complex flow process of knowledge and technology that involves various influencing factors. Therefore, this also leads to the diversification of research perspectives. Combing relevant literature reveals the research is mainly conducted from the following two perspectives. First, the human capital perspective. Human capital is an important foundation for realizing FDI technology spillovers. The level of human capital determines a region’s ability to learn, to absorb and to innovate, to a certain extent. Relevant studies such as Osei and Kim (2020) have found that in the initial and development stage of FDI, the contribution of higher education increases gradually, while that of secondary education gradually decreases. Nie et al. (2021) examined the principle of action of FDI spillover effects based on the path of human capital flow and argue that FDI has a significant effect of capital accumulation on China’s economic growth. Constrained by the degree of financial development, the stimulation effects of China’s FDI technology and human capital spillovers on the domestic economy are still not obvious. Tuzun and Kalemci (2017) included human resources in the scope of their research. They established a convergence model of economic growth on the basis of neoclassical growth theory and found that FDI and human capital factors provide a strong push to regional economic growth. Second, the perspective of environmental regulations. On one hand, reasonable environmental regulations can effectively filter out foreign investments that produce high levels of pollution and high emissions and reduce the entry of such investments at source. On the other hand, environmental regulations can encourage FDI enterprises to strengthen their technological innovation and R&D investment, which will be conducive to technology spillovers. However, stronger environmental regulations will crowd out investments in innovation and R&D, overwhelm the enterprises, and are not conducive to FDI technology spillovers and the enhancement of innovation and learning capabilities of local enterprises. Hu et al. (2018) argues that environmental policy games exist between local governments in China due to the competition for FDI, but the effect of environmental regulations on FDI is not significant on average. Using a game theory framework of a three-stage imperfectly competitive market, Ouyang et al. (2020) investigated the impact of environmental regulations and corruption on FDI inflows, and the result supported the “pollution haven” hypothesis. Du et al. (2019) argue that the intensity of environmental regulations affects the entry mode of FDI and has a differential effect on the efficiency of green technology innovation in regions that FDI enters. Through joint ventures and sole proprietorship, FDI enters areas with stronger and weaker environmental regulations, respectively. It is further revealed that when joint ventures enter areas with stronger environmental regulations, it is not conducive to the enhancement of the green technology innovation efficiency. When sole proprietorship enters areas with weaker environmental regulations, it will effectively improve the green technology innovation efficiency. Furthermore, there are the perspectives of industrial linkage, industry heterogeneity, and competitions between local governments.

This study draws on the results of previous studies, but there are still few that examine FDI technology spillovers based on the level of marketization. As the construction of the market economy is imperfect, the factor market, product market, and legal environment of China are all distorted to varying degrees, which severely restricts the effective allocation of resources. “The Decisive Role of the Market in Resource Allocation” proposed by the 3rd Plenary Session of the 18th CPC Central Committee provided a clear direction for further improving the construction of the market economy. In May 2020, the “Opinions of the CPC Central Committee and the State Council on Accelerating the Improvement of the System of Socialist Market Economy in a New Era” was published. These clearly stated that the focus should be on improving the market-based allocation of factors, to accelerate the construction of a market system that is unified, open, and with orderly competition, and to enhance the construction of a factor market system. The opinions were also introduced to ensure market-oriented pricing for factors, free and orderly flow of factors, and efficient and fair allocation of factors. Therefore, examining the effect of FDI technology spillover on carbon emissions from the perspective of marketization level will provide new ideas for making better use of FDI technology spillover, formulating effective policies for introducing foreign investment, and deepening the construction of regional marketization in the new era. The major marginal contributions of this study are as follows. First, the research perspective of marketization level is different from existing perspectives such as financial development, human capital, and environmental regulation, and provides new ideas for the research of FDI technology spillovers. Second, unlike traditional research on linear relationships, a non-linear relationship may exist in the effect of FDI on carbon emissions under different levels of marketization. The use of a threshold model will contribute to discover such a relationship and reveal the influence relationships between the variables with higher accuracy. Third, the low-carbon construction of the Yangtze River Economic Belt is of great significance to achieving China’s overall low-carbon goals, but there are still few academic achievements on the low-carbon research of the provinces and cities of this area. The current study helps to enrich the results in such research. The rest of this paper is organized as follows. In section, we review the relevant literature and discuss the content of our study. In section “Threshold mechanism of marketization level in the effect of FDI on carbon emissions” presents the analysis of the mechanism of marketization level in the effect of FDI on carbon emissions. Section “Indicator selection and model construction” partially explains the selection of indicators and construction of the model, while section “Empirical results and analysis” is an analysis of empirical results and the robustness test. Finally, section “Research conclusions and policy suggestions” presents the research conclusions and policy implications.

## Threshold Mechanism of Marketization Level in the Effect of FDI on Carbon Emissions

**FIGURE 1:**
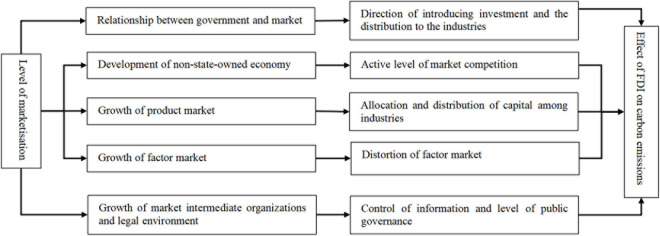
Level of marketisation and its mechanism in the effect of FDI on carbon emissions.

Cole and Fredriksson (2009) proposed that a good market environment is conducive to FDI technology spillovers, the acceleration of the enhancement of local resource utilization efficiency, and the gradual improvement of the local environmental quality. The level of marketization mainly covers five aspects: the relationship between the government and the market, the development of the non-state-owned economy, the growth of the product market, the growth of the factor market, the growth of market intermediary organizations, and the legal system environment (Millimet and Roy, 2016). The five aspects all play a role in the effect of FDI on carbon emissions from different perspectives, as shown in Figure 1. In particular, the relationship between the government and the market; at this stage, China still adopts a strong government-led market economy. Local governments exert compelling guiding influence on the economic development of their own regions, while such influence is more significant in the central and western regions, which are economically underdeveloped. In the context of information asymmetry and unreasonable assessment, the major effects of the government’s direct intervention in economic activities include the following. First, the government determines the direction of introducing investment and the distribution to the industries. The pursuit of short-term political achievements, unreasonable assessment systems, and promotion of officials of the local governments induce weak implementation of environmental regulations, so that foreign enterprises that produce high levels of pollution and high emissions enter easily, which undoubtedly intensifies the pressure on regional carbon emissions reduction. Second, as local governments take the initiative to push down the resource prices such as land and labor, enterprises carry out extensive scale expansion by expanding the input of traditional resources, resulting in a crowding-out effect on innovation activities, and a lack of innovation consciousness of enterprise, which is unfavorable to the enhancement of resource utilization efficiency. *Development of non-state-owned economy*: The proportion of non-state-owned economy in the regional economy reflects the active level of market competition to a certain extent. Different from the soft budget constraints of state-owned enterprises, non-state-owned enterprises face hard budget constraints and can rapidly respond to market changes, have stronger initiative to innovate and change, and have higher operational efficiency. At the same time, the market competition brought by the development of the non-state-owned economy also boosts the hardening of the budget constraints of the existing state-owned enterprises (Schümann et al., 2021). *The degree of growth of the product market*: The degree of growth of the product market is measured specifically from two aspects. First, the degree of effect of the market on price determination, and second, the degree of influence that local protection exerts on the product market. The greater the degree of effect of the market on commodity prices, the lower the local protection, and more mature the growth of the product market. *The degree of growth of the factor market*: The factor market is closely related to the commodity market, and the latter guides the mobilities of various factors. The factor market covers the financial, property, labor, and land markets. The higher the degree of growth, the more timely and accurate the reflection of market demand and supply information. At present, governments at all levels still have too much power in the control of production factors. For example, a series of regulations on household registration restrict the mobility of labor factors. The profit-seeking nature of FDI makes it extremely sensitive to the commodity and factor markets in the target region. At present, different factor price distortions exist in the factor markets of various regions of China. For instance, to attract more foreign investment, local governments take the initiative to lower the prices of labor factors. Such approaches to control factor prices have a significant effect in the short run and can realize rapid enhancement of economic growth. However, it is not conducive to economic and social development in the long run. The main reason is that cheap labor induces enterprises’ willingness to use tangible factors, and lowers their eagerness to innovate and change. In the long run, as the labor dividend vanishes, while the innovation level has not been effectively promoted, the regional economies will face serious consequences. Cheng et al. (2020) and Liu et al. (2016) have proven this viewpoint. *The growth of market intermediary organizations*: Market intermediary organization is a third organization in addition to the government and the market, which exerts an important intermediary effect when the latter two are defective. Such organizations provide a certain degree of social services and consultation information to different parties. At present, those in China are still imperfect, in terms of low independence of intermediary organizations, insufficient coverage of different categories, and low quality of operations. The immature growth of such organizations affects the enterprises’ control of various information, including those of the market and the government, as well as the resource allocation efficiency. *Legal system environment*: The legal environment reflects the level of regional public governance, and the activities of the market economy rely on the protection of the legal environment. Areas that are plagued by rent-setting, rent-seeking, and show no respect for contracts discourage the agglomeration of high-quality resources, let alone effective knowledge and technology spillover.

Comprehensive analysis reveals that, in terms of the environment of the target area FDI enters as well as the business environment, the level of marketization plays a role in the effect of FDI on carbon emissions. However, the direction and magnitude of the influence cannot be accurately measured based on qualitative analysis, and it is necessary to combine qualitative and quantitative analyses to obtain a comprehensive grasp of the influence level of the level of marketization.

## Indicator Selection and Model Construction

### Indicator Selection

#### Explained Variable

The major indicators for measuring carbon emissions performance include the total- and the single-factor carbon emissions efficiency indexes. As a crucial single-factor carbon emissions efficiency index, carbon emissions intensity has become one of the main measurement indicators in pursuit of the international target of emissions reduction, while it also reflects that development is the top priority of contemporary China. China set forth a target to reduce carbon emissions intensity by 60–65% by 2030, with 2005 as a benchmark. The target has been incorporated into the Plan for National Economic and Social Development as a binding index. Therefore, to measure carbon emissions performance with carbon intensity has strong practical significance, which also constitutes the starting point of theoretical analysis (Liu et al., 2016). Carbon emissions intensity is measured by the ratio of total carbon emissions to gross regional product, denoted by *CEI*.

#### Core Explanatory Variable

The annual actual use of foreign capital of the provinces and cities is adopted as the FDI. The indicator is divided into stock and flow. The flow indicator ignores the effect of FDI accumulation in the previous period on the current period. Therefore, the stock indicator is used to construct the core explanatory variable, while the flow indicator is used for the robustness test. As the statistical yearbooks and data do not reveal the exact FDI stocks in provinces and cities, this study follows Yao and Wei (2007) and uses the perpetual inventory method (PIM) for the estimation; the calculation is as shown in Equation 1:


(1) $${\mathrm{FDI}}_{i,t}^{s}={\mathrm{FDI}}_{i,t-1}^{s}\,\left(1-\delta_{i,t}\right)+{\mathrm{FDI}}_{i,t}$$

where $F\,D\,I_{i,t}^{\text{s}}$ and $F\,D\,I_{i,t-1}^{\text{s}}$ are the FDI stocks of region *i* during the periods of *t* and *t*−1, respectively. *FDI*_i,t_ is the actual use of foreign capital of region *i* during period *t*. δ_i,t_ is the depreciation rate, but the selection of δ value is still controversial in academia. According to the PIM, strictly speaking, δ refers to the replacement rate of capital necessary to maintain relatively constant production conditions, but not the depreciation rate. Only when the relative efficiency of capital is geometrically decreasing are the two values equal. If the difference between the two is ignored, different δ values will be generated (Li et al., 2018). Dombi (2018) conformed with the important principle and considered the connotation of perpetual inventory. Under the assumption that the relative efficiencies of the capital goods are geometrically decreasing, the author adopted the declining balance method, which represents geometrically decreasing efficiency accordingly, and estimated that the δ value was 9.6%. After the relevant research results are weighed, the current study adopts the above estimation result and takes 9.6% as the δ value. The ratio of $F\,D\,I_{i,t}^{\text{s}}$ to *GDP*_i,t_ is taken as the foreign participation and is the core explanatory variable, which is denoted as *FDI1*. The ratio of *FDI*_i,t_ to *GDP*_i,t_ is denoted as *FDI2*, which is used for the robustness test.

#### Threshold Variable

The level of marketization is the threshold variable. After combing through the academic achievements of predecessors, it is revealed that there are different standards of measuring the levels of marketization. For example, Lucey et al. (2020) represent the level with the ratio of the employed population in non-state-owned economy to the total employed population. Zang et al. (2020) measured the level with the ratio of local fiscal expenditure to GDP. Chen (2012) constructed a marketization level index system for a principal components analysis to measure the level of marketization of provinces and cities. The level of marketization is a comprehensive index that measures regional economic systems. If the measurement of the level is limited to a single indicator, then a large amount of information will be missed. This study draws on Yu and Wang (2016) marketization index to measure the level of marketization. Since the data in the report cover the years 2008–2014, the data of 2015–2016 are estimated using trend extrapolation and is denoted as *Market*.

#### Other Control Variables

There are many other factors that affect carbon emissions intensity. Based on existing research and the availability of data, this study includes the following types of main influencing factors. First, the energy structure. China has an energy resource structure that is “rich in coal but poor in oil and gas.” This characteristic is an important reason for the imbalance of the energy consumption structure. The huge consumption of traditional fossil energy results in more pressure on carbon reduction. The proportion of coal consumption in total energy consumption is used to represent the energy structure, which is denoted as *ES*. Second, energy intensity, which reflects the efficiency of regional energy use. It is represented by the ratio of total energy consumption to regional GDP, which is denoted as *EI*. Third, industrial structure. The secondary industry is that with the largest carbon emissions. At this stage, the proportion of the secondary industry in each province or city largely determines the scale of carbon emissions in the region. The industrial structure is represented by the proportion of the production value of the secondary industry in regional GDP, and is denoted as *IS*. Fourth, opening to the outside world. Regions with higher openness to the outside world can learn and absorb advanced production technology as well as management experience at home and abroad more quickly and increase the energy utilization efficiency. The openness to the outside world is represented by the ratio of the total export-import volume of a region to GDP and is denoted as *Open*. Fifth, technological innovation. The measurement of the level of technological innovation draws on the method of Foder et al. (2008) and is represented by the number of patent applications in each region, which is denoted as *Tech*. Sixth, urbanization. The advancement of urbanization is accompanied by a large number of infrastructure constructions, and increases the demand for industrial products. The Yangtze River Economic Belt, especially in its middle and upper reaches, is still in a stage of acceleration in terms of urbanization, while the level and quality of urbanization has significant room for improvement. The level of urbanization is represented by the proportion of urban residents in the year-end permanent residents of each region and is denoted as *Urb*.

### Panel Threshold Model

In threshold studies, the existing academic results are mainly achieved using group testing and models of interaction terms. In group testing, the cut points are set with subjective experience. On the other hand, the testing of interaction terms is constrained by the uncertainty of the form of the terms. Neither of the methods can serve the purpose of performing significance tests on threshold effects. The threshold regression model proposed by Liu et al. (2021) can overcome the shortcomings of the above two methods. In addition to providing accurate estimation of the threshold values, it can also complete the significance test. Therefore, this method is used to construct a threshold model of the effect of FDI on carbon intensity, which takes level of marketization as the threshold variable, as shown in Equation 2.


$${\mathrm{CEI}}_{i,t}=c+\beta_{1}{\mathrm{FDI}}_{i,t}*I\left({\mathrm{Market}}_{i,t}<\gamma \right)$$

$$+\beta_{2}{\mathrm{FDI}}_{i,t}*I\left({\text{Market}}_{i,t}\geq \gamma \right)+\alpha_{1}{\mathrm{ES}}_{i,t}+\alpha_{2}{\mathrm{ES}}_{i,t}+\alpha_{3}{\mathrm{ES}}_{i,t}$$

(2) $$+\alpha_{4}\,{\mathrm{Open}}_{i,t}+\alpha_{5}\,{\mathrm{Tech}}_{i,t}+\alpha_{6}\,{\mathrm{Urb}}_{i,t}+\delta_{i}+\xi_{i,t}$$

Equation 2 presents the single-threshold model of the level of marketization in the effect of FDI on carbon emissions intensity, and the multi-threshold model can be expanded and obtained *via* the same approach. *Market* is the threshold variable, γ is the threshold value, *I*(⋅) is an indicator function, that is, when the expression in the bracket is true, its value is 1, otherwise 0. c and ξ_i,t_ are the constant and the residual, respectively, and δ_i_ is the fixed effect of non-observed areas. The calculation of the threshold model is mainly divided into the following two stages. First, conduct a parameter estimation of the threshold value and that of the related control variables, whose estimation is mainly done by minimizing the OLS estimated residual under the assumed number of thresholds. Second, conduct a significance test of the estimated parameters. Specifically, the panel data {*y*_i,t_,*x*_i,t_,*q*_i,t_:1≤*i*≤*n*,1≤*t*≤*T*} is determined, where *i* denotes an individual and *t* denotes the time. Liu et al. (2021) proposed the following fixed-effect threshold model:


(3) $$\left\{ \begin{array}{c} y_{i,t}=\mu_{i}+\beta_{1}^{\prime }\,{\text{x}}_{i,t}+\varepsilon_{i,t},\mathrm{if}\;q_{i,t}\leq \gamma \\ y_{i,t}=\mu_{i}+\beta_{2}^{\prime }\,{\text{x}}_{i,t}+\varepsilon_{i,t},\mathrm{if}\;q_{i,t}>\gamma \end{array}\right.$$

where *q*_i,t_ is the threshold variable, γ is the threshold value to be estimated, and ε_i,t_ is independent and identically distributed. With the indicator function, Equation 3 can be further simplified into the following form:


(4) $$y_{i,t}=\mu_{i}+\beta_{1}^{\prime }\,x_{i,t}\cdot {\mathrm{Iq}}_{i,t}\leq \gamma +\beta_{2}^{\prime }\,x_{i,t}\cdot {\mathrm{Iq}}_{i,t}>\gamma +\varepsilon_{i,t}$$

Let $\text{β}\equiv \left(\begin{array}{c} {\text{β}}_{1}\\ {\text{β}}_{2} \end{array}\right)\;x_{i,t}(\text{γ})\equiv \left(\begin{array}{c} x_{i,t}\cdot 1q_{i,t}\leq \text{γ}\\ x_{i,t}\cdot 1q_{i,t}>\text{γ} \end{array}\right)$, thus, Equation 4 can be further simplified into the following form:


(5) $$y_{i,t}=\mu_{i}+\beta^{\prime }\,{\text{x}}_{i,t}\,\left(\gamma \right)+\varepsilon_{i,t}$$

Average the time on both sides of Equation 5 to obtain Equation 6, and subtract Equation 6 from Equation 5 to obtain the deviation form, as in Equation 7:


(6) $${\bar{y}}_{i}=\mu_{i}+\beta^{\prime }\,{\bar{x}}_{i}\,\left(\gamma \right)+{\bar{\varepsilon }}_{i}$$

(7) $$y_{i,t}-{\bar{y}}_{i}=\beta^{\prime }\,\left[x_{i,t}\,\left(\gamma \right)-{\bar{x}}_{i}\,\left(\gamma \right)\right]+\left(\varepsilon_{i,t}-{\bar{\varepsilon }}_{i}\right)$$

Let $y_{i,t}^{*}\equiv y_{i,t}-{\bar{y}}_{\text{i}}$, $x_{i,t}^{*}\equiv x_{i,t}\,\left(\gamma \right)-{\bar{x}}_{\text{i}}\,\left(\gamma \right)$, $\varepsilon_{i,t}^{*}\equiv \varepsilon_{i,t}-{\bar{\varepsilon }}_{\text{i}}$; Equation 8 can be obtained:


(8) $$y_{i,t}^{*}=\beta^{\prime }\,x_{i,t}^{*}\,\left(\gamma \right)+\varepsilon_{i,t}^{*}$$

First, the value of γ is given, while OLS is used for the consensus estimation of Equation 8, to obtain the estimated coefficients $\hat{\beta }\,\left(\gamma \right)$ and residual sum of squares SSR (γ). Next, for γ ∈ {*q*_i,t_:1≤*i*≤*n*,1≤*t*≤*T*}, $\hat{\gamma }$ is chosen to obtain a minimized value of SSR ($\hat{\gamma }$) and the estimated coefficients $\hat{\beta }$ ($\hat{\gamma }$).

To determine whether there is a threshold effect, the following null hypothesis can be tested: *H*_0_:β_1_ = β_2_. If the null hypothesis holds, there is no threshold effect. If *H*_0_:β_1_ = β_2_ is rejected, it is considered that there is a threshold effect, and the threshold value can be further tested, that is, to test *H*_0_:γ = γ_0_. Define the likelihood ratio test statistic as $\text{LR}\,\left(\gamma \right)\,\left[S\,S\,R\,\left(\gamma \right)-S\,S\,R\,\left(\hat{\gamma }\right)\right]/{\hat{\sigma }}^{2}$, and it can be proved that in the case of *H*_0_:γ = γ_0_ holds, although the asymptotic distribution of LR(γ) is still non-standard, its cumulative distribution function is (1−*e*^−x/2^)^2^, and its critical values can be directly calculated. Therefore, statistics LR(γ) can be used to calculate the confidence intervals. In the same way, the multi-threshold situation can also be calculated using this method.

### Carbon Emissions Accounting and Data Sources

#### Carbon Emissions Calculations

As the amounts of carbon emissions of the provinces and cities are not directly revealed in different statistical yearbooks, the numbers must be estimated based on the final consumptions of various types of energy. With reference to the calculation methods and related research results published by the Ipcc. (2006), the following calculation equation is adopted. Consider Equation 9:


(9) $$\mathrm{CE}=\sum_{i}^{14}F_{i}\times {\mathrm{CV}}_{i}\times {\mathrm{CCF}}_{i}\times {\mathrm{COF}}_{i}\times \left(\frac{44}{12}\right)$$

where CE denotes carbon emissions (10,000 tons), and *i* represents the type of energy. The final consumptions of 14 types of energy, including raw coal, washed coal, other washed coals, coke, coke oven gas, other coal gases, crude oil, gasoline, paraffin, diesel, fuel oil, liquefied petroleum gas, refinery dry gas, and natural gas are selected. *F* is the final fossil energy consumption (10,000 tons or 100 million cubic meters), *CV* is the average lower calorific value (kJ/kg or kJ/m^3^), *CCF* is the carbon content of various energy (kg/billion joule), *COF* is the carbon oxidation rate; 44 and 12 denote the molecular mass of carbon dioxide and the atomic mass of carbon, respectively.

#### Data Sources

Considering the availability of data and the practical research, the study period covers 2008–2016. For the relevant indicators involving price factors, the indicators are all deflated to 2008 prices based on the relevant price index to reduce the impact of inflation and other factors on the data. At the same time, logarithmic processing is also performed to reduce the impact of data fluctuation and heteroscedasticity. According to geographical location, the Yangtze River Economic Zone is divided into eastern, central, and western regions. The eastern region includes Shanghai City, Jiangsu, and Zhejiang provinces. The central region includes Anhui, Jiangxi, Hubei, and Hunan provinces, and the western region includes Chongqing City, Sichuan, Guizhou, and Yunnan provinces. All data come from the “China Statistical Yearbook,” “China Energy Statistical Yearbook,” “China Compendium of Statistics 1949–2008,” and the “Statistical Yearbook” of 11 provinces and cities in the Yangtze River Economic Belt, and the provincial statistical database of the National Bureau of Statistics. The descriptive statistics of the indicators are presented in Table 1.

**TABLE 1:** Descriptive statistics of variables.

	**Variable**	**Meaning**	**Mean**	**Standard deviation**	**Maximum**	**Minimum**
Explained variable	lnCEI	Carbon emission intensity	0.2517	0.4522	1.1741	–0.5756
Core explanatory variable	lnFDI1	Foreign participation	–1.7398	0.6595	–0.6595	–3.0889
Control variables	lnES	Energy structure	–0.5043	0.3177	–0.1699	–1.5646
	lnEI	Energy intensity	–0.0005	0.3225	0.7422	–0.4912
	lnIS	Industrial structure	–0.7721	0.1256	–0.5912	–1.2096
	lnOpen	Openness to the outside world	–1.6814	0.9857	0.4636	–3.4374
	lnTech	Technological innovation level	9.5723	1.2530	12.0847	6.8985
	lnUrb	Urbanization rate	–0.6719	0.2580	–0.1097	–1.2339
Threshold variable	lnMarket	Marketization level	6.8951	1.7525	10.8984	3.5500

## Empirical Results and Analysis

### Basic Overview of the Yangtze River Economic Belt

The 11 provinces and cities of the Yangtze River Economic Belt include Shanghai, Jiangsu, Zhejiang, Anhui, Jiangxi, Hubei, Hunan, Chongqing, Sichuan, Yunnan, and Guizhou, covering an area of approximately 2.05 million square kilometers, with a population and GDP exceeding 40% of that of the country. In 2016, the “Guidelines for Development Along the Yangtze Economic Belt” was released, formulating a development pattern of “one axis, two wings, three poles, and multiple points.” At a seminar on the development of the Yangtze Economic Belt in 2016, it was proposed that the restoration of the ecological environment of the Yangtze River is of utmost importance, while joint efforts must be devoted to major plans of protection instead of development. This provides a clear direction for the economic development and ecological construction of the Yangtze River Economic Belt.

### Carbon Emissions Intensity, Foreign Participation, and Marketization Level in the Yangtze River Economic Zone

**FIGURE 2:**
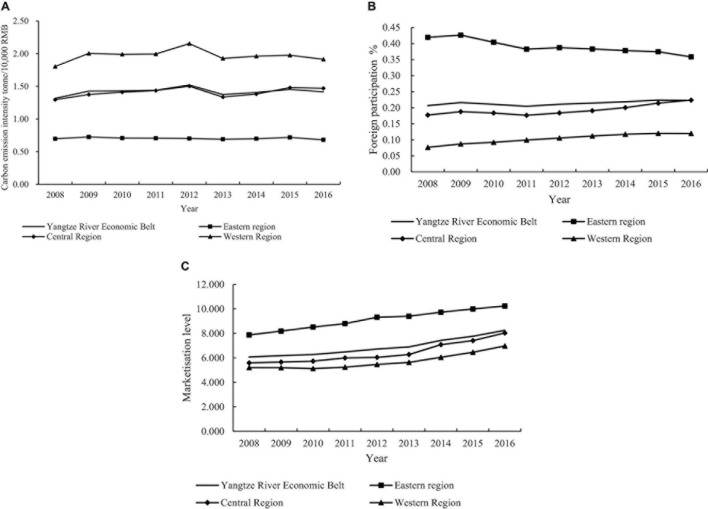
**(A)** Carbon emissions intensities of Yangtze River Economic Belt and regions (2008–2016). **(B)** Foreign participation of Yangtze River Economic Belt and regions (2008–2016). **(C)** Marketisation level of Yangtze River Economic Belt and regions (2008–2016).

From Figure 2A, it can be concluded that carbon emissions intensity shows a pattern of western region > central region > eastern region. The carbon emissions intensities of regions show significant differences, while the overall intensity of the Yangtze River Economic Belt is close to that of the central region.

From Figure 2B, it can be concluded that the foreign participation in the eastern region decreases and fluctuates. The main reasons are that traditional cost advantages decrease as the prices of factors such as labor and land have increased in the eastern region. Parts of the foreign capitals originally invested in the eastern region gradually flow to the central and western regions as well as Southeast Asia. The central and western regions actively undertake the transferred industries, and the foreign participation shows a steady upward trend.

From Figure 2C, it can be concluded that the marketization level of the eastern, central, and western regions of the Yangtze River Economic Belt all show a steady upward trend. The marketization level of the eastern region is significantly higher than that of the central and western regions, while the difference between the central and western regions is smaller.

### Analysis of the Panel Threshold Model

Stata 14.0 was used in the analysis. According to the solution of the panel threshold model proposed by Liu et al. (2021), a test was first performed to confirm whether there was a threshold effect. The threshold effect test results obtained by 300 times of bootstrap sampling are reported in Table 2.

**TABLE 2:** Panel threshold test results.

**Threshold variable**	**Number of thresholds**	***F*-value**	***P*-value**	**10%**	**5%**	**1%**
lnMarket	Single threshold	40.15***	0.0000	19.9656	22.0518	26.7072
	Double threshold	17.29*	0.0833	16.5047	18.7378	23.2747
	Triple threshold	15.03	0.7500	45.4456	52.6723	65.5581

The symbols *** and * denote significance at the 1 and 10% critical valuelevels, respectively. Both the *P*-value and the critical value are obtained by repeated sampling for 300 times using the bootstrap method.

**FIGURE 3:**
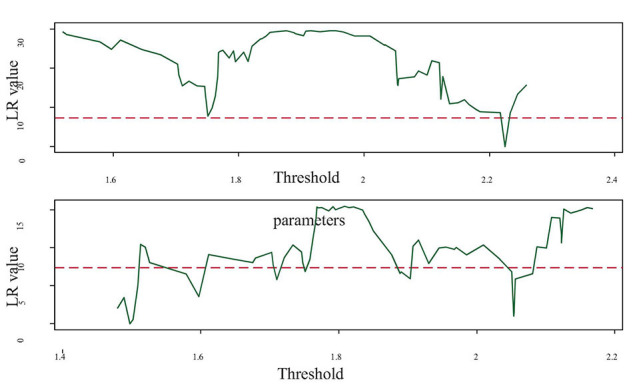
Likelihood ratio function graph of threshold effect.

Table 3 and Figure 3 are the threshold estimation and likelihood ratio function graph, respectively. The threshold estimates are the values when the likelihood ratio statistic *LR* is 0, therefore, the distribution of the double threshold is 1.4974 and 2.2248. The threshold regression estimation results are further obtained, as presented in Table 4.

**TABLE 3:** Threshold estimates and confidence intervals.

**Threshold variable**	**Threshold value**	**Threshold estimate**	**95% confidence interval**	
lnMarket	γ1	1.4974	1.4888	1.5019
	γ2	2.2248	2.217	2.2332

**TABLE 4:** Regression results of double threshold effect.

**Variable**	**Coefficient estimate**	***t*-value**	***P*-value**
lnES	0.1667***	3.36	0.0010
lnEI	1.2490***	21.83	0.0000
lnIS	0.5537***	5.62	0.0000
lnOpen	−0.1599***	–10.11	0.0000
lnTech	−0.0567***	–5.65	0.0000
lnUrb	0.2154**	2.60	0.0110
lnFDI1*I (lnMarket <1.4974)	0.2530***	6.05	0.0000
lnFDI1*I (1.4974 < lnMarket < 2.2248)	0.2026***	5.50	0.0000
lnFDI1*I (lnMarket >2.2248)	0.1683***	4.95	0.0000
Constant	0.6675***	4.61	0.0000

The symbols *** and ** denote significance at the 1 and 5% critical valuelevels, respectively.

The threshold test results in Table 2 indicate that, when the marketization level is taken as the threshold variable, the assumptions of the single and double thresholds are significant at the significance level of 1 and 10%, respectively, but the triple threshold did not pass the significance test. Therefore, it can be concluded that the threshold model has a double threshold effect.

From the results in Table 4, when lnMarket < 1.4974 (i.e., Market < 4.4701), the coefficient of foreign participation is 0.2530, and is significant at the 1% critical value level. It indicates that when Market < 4.4701, foreign participation increases by 1%, and carbon emissions intensity increases by 0.2530%. When 1.4974 < lnMarket < 2.2248 (i.e., 4.4701 < Market < 9.2516), the coefficient of foreign participation is 0.2026. When lnMarket > 2.2248 (i.e., Market > 9.251), the coefficient of foreign participation is 0.1683, while both values are significant at the 1% critical value level. From the perspective of different marketization levels, the coefficients of foreign participation are all positive, indicating that FDI increases carbon emissions intensity. In general, with the promotion of marketization, the regression coefficient of foreign participation gradually decreases, and falls from 0.2530 to 0.1683, but it does not become negative. The spillover of FDI technology does not offset the corresponding pollution effect. The overall marketization level of the Yangtze River Economic Zone is still weak, especially in the provinces and cities of the central and western regions, where the promotion of marketization level is slow. Weaker levels of marketization suppress the level of FDI technology spillovers from multiple dimensions, and the Yangtze River Economic Belt is reduced to a “pollution haven” for foreign investment. Table 5 presents the statuses of how the marketization levels of the eleven provinces and cities in the Yangtze River Economic Belt cross the thresholds, in 2008–2016.

**TABLE 5:** Statuses of how the marketization levels of the 11 provinces and cities in the Yangtze River Economic Belt crossed the threshold, 2008–2016.

**Year**	**Market < 4.4701**	**4.4701 < Market < 9.2516**	**Market > 9.2516**
2008	Guizhou	Shanghai, Jiangsu, Zhejiang, Anhui, Jiangxi, Hubei, Hunan, Chongqing, Sichuan, and Yunnan	Nil
2009	Guizhou	Shanghai, Jiangsu, Zhejiang, Anhui, Jiangxi, Hubei, Hunan, Chongqing, Sichuan, and Yunnan	Nil
2010	Guizhou	Shanghai, Jiangsu, Zhejiang, Anhui, Jiangxi, Hubei, Hunan, Chongqing, Sichuan, and Yunnan	Nil
2011	Guizhou	Shanghai, Jiangsu, Zhejiang, Anhui, Jiangxi, Hubei, Hunan, Chongqing, Sichuan, and Yunnan	Nil
2012	Guizhou	Shanghai, Anhui, Jiangxi, Hubei, Hunan, Chongqing, Sichuan, and Yunnan	Jiangsu and Zhejiang
2013	Nil	Shanghai, Anhui, Jiangxi, Hubei, Hunan, Chongqing, Sichuan, Guizhou, and Yunnan	Jiangsu and Zhejiang
2014	Nil	Anhui, Jiangxi, Hubei, Hunan, Chongqing, Sichuan, Guizhou, and Yunnan	Jiangsu, Zhejiang, and Shanghai
2015	Nil	Anhui, Jiangxi, Hubei, Hunan, Chongqing, Sichuan, Guizhou, and Yunnan	Jiangsu, Zhejiang, and Shanghai
2016	Yunnan	Anhui, Jiangxi, Hubei, Hunan, Sichuan, and Guizhou	Jiangsu, Zhejiang, Shanghai, and Chongqing

With 2012 as the time node, in 2008–2011, except for Guizhou Province, all remaining provinces and cities lay between the first and second thresholds, while none successfully crossed the second threshold. In 2012–2016, the marketization levels of Jiangsu and Zhejiang provinces were the first to cross the second threshold, while Guizhou Province gradually crossed the first threshold and entered the ranks between the first and second thresholds. In 2016, Chongqing became the only region in the central and western regions that successfully crossed the second threshold. On the whole, the central and western regions are still firmly stuck between the first and second thresholds due to the relatively weak economic foundation and the relatively slow marketization process. The eastern region opened to the outside world earlier, has a better economic foundation, superior geographic location, as well as a high mobility of factors, and the level of marketization is higher. Since the 18th National Congress of the Chinese Communist Party, especially since the 3rd Plenary Session of the 18th CPC Central Committee, governments at all levels have further sought to clarify the relationship between the government and the market, vigorously simplified the administrative procedures and delegated the powers, combated corruption, deepened the “decentralization-control-service” reform, and exerted the decisive role of the market in resource allocation. The deepening of reform has achieved certain results. However, to realize the target of pushing all 11 provinces and cities in the Yangtze River Economic Belt across the second thresholds, or even realize the suppression effect of FDI on carbon emissions, it is still necessary for all regions to accelerate the construction of regional marketization and the steps of various reform plans continuously.

For other control variables, the coefficients of energy structure and energy intensity are 0.1667 and 1.2490, respectively, which are both significant at the 1% significance level, indicating that carbon emissions intensity increases. Among them, energy intensity has become the most important factor that affects carbon emissions intensity. Energy intensity is an important manifestation of regional economic benefits and technological level. Reducing the energy intensity is of great significance to the implementation of low-carbon development. The constraints of energy resource endowment make it difficult for the energy structure to have major changes in the short run. Distorted energy prices cannot reflect the actual environment costs that it causes and increase the consumption of traditional fossil energy. The formulation of effective energy industry policies, development of new and clean energy industries, and the enlargement of the proportions of such energy are undoubtedly strong guarantees for the realization of clean production. The coefficient of industrial structure is 0.5537, and it is significant at the 1% level, that is, when the industrial structure increases by 1%, the carbon emissions intensity will increase by 0.5537%. One of the main targets of the Yangtze River Economic Belt is to establish itself as an advanced center of equipment manufacturing. Decreasing the carbon emissions intensity does not mean to decrease the proportion of the second industry in terms of the number and to pursue a superficial industrial relationship, rather, it means to phase out backward production capacities, increase the efficiency of resource allocation, ensure continuous flow of resource factors to industries with higher efficiency, and to promote the levels of industrial upgrades and rationalization. The coefficients of openness to the outside world and technological innovation are −0.1599 and −0.0567, respectively, and are significant at the 1% level, indicating that increasing these factors is conducive to reducing carbon emissions intensity, and it is in line with economic expectations. The increase in the openness is of great significance for strengthening economic ties with the outside world, as well as for learning and absorbing advanced technology and management experience. The central and western regions of the Yangtze River Economic Belt still have lower openness to the outside world. In the future, full use should be made of the advantages of the Yangtze River’s “golden waterways” and the openness should continue to be expanded. The coefficient of urbanization is 0.2154 and is significant at the 5% level. A large number of infrastructure constructions will inevitably arise with the continuous advancement of urbanization and induce the consumption of energy-intensive and high-emission products such as cement, steel, and building materials. When the focus of urbanization upgrades and transforms from quantity to quality improvement, the industrial, technology, and talent agglomeration brought about by urbanization will gradually produce effective spillovers, and in turn increase the utilization efficiency of regional resource and reduce the carbon emissions intensity. The three provinces and cities in the eastern region already have relatively high levels of urbanization rates, while a large gap remains between the central and western regions and the eastern region. All regions should make more efforts in terms of rational planning and avoid blindly pursuing scale expansion and neglecting the quality enhancement of urbanization development.

### Robustness Test of the Panel Threshold Model

The previous sections introduced a threshold model that uses FDI stock to construct indicators of foreign participation and investigates the effect of FDI on carbon emissions intensity under different marketization levels. It is necessary to test the robustness of the empirical results. With all other variables unchanged, we replace FDI stock with FDI flow, construct foreign participation indicator *FDI2*, and test the robustness of the abovementioned threshold model. The exact results are presented in Tables 6–8.

**TABLE 6:** Panel threshold test results.

**Threshold variable**	**Number of thresholds**	***F*-value**	***P*-value**	**10%**	**5%**	**1%**
lnMarket	Single threshold	32.63**	0.0367	22.5437	28.2682	51.2541
	Double threshold	22.37*	0.0533	18.1154	23.2467	33.5832
	Triple threshold	6.43	0.9100	40.9802	44.6350	54.0601

The symbols ** and * denote significance at the 5 and 10% critical valuelevels, respectively.

**TABLE 7:** Threshold estimation and confidence intervals.

**Threshold variable**	**Threshold value**	**Threshold estimate**	**95% confidence interval**	
lnMarket	γ1	1.6752	1.6281	1.6790
	γ2	2.2248	2.1925	2.2332

**TABLE 8:** Regression results of double threshold effect.

**Variable**	**Coefficient estimate**	***t*-value**	***P*-value**
lnES	0.3174***	7.34	0.0000
lnEI	1.2919***	26.56	0.0000
lnIS	0.7013***	7.41	0.0000
lnOpen	−0.1115***	–6.29	0.0000
lnTech	−0.0599***	–6.34	0.0000
lnUrb	0.3995***	5.63	0.0000
lnFDI2*I (lnMarket < 1.6752)	0.1742***	7.80	0.0000
lnFDI2*I (1.6752 < lnMarket < 2.2248)	0.1486***	7.31	0.0000
lnFDI2*I (lnMarket > 2.2248)	0.1082***	6.05	0.0000
Constant	0.9829***	6.90	0.0000

The symbols *** denote significance at the 1% critical valuelevels.

From the threshold test and threshold value estimation results in Tables 6, 7, the robustness threshold test also concludes that a double threshold exists, and the threshold values are 5.3398 and 9.2516, respectively. The results in Table 8 indicate that the coefficients of foreign participation in the three threshold intervals are 0.1742, 0.1486, and 0.1082, respectively, and are significant at the 1% critical value level. Compared with the threshold model structure obtained using the FDI stock, the signs of coefficients in the robustness regression results are basically consistent with the previous sections, but there are slight differences in the magnitudes of the coefficients. It can therefore be concluded that the regression analysis of the threshold model, which investigates the effect of FDI on carbon emissions intensity from the perspective of marketization levels, are robust, and the relevant conclusions are reliable.

## Research Conclusion and Policy Suggestions

### Research Conclusion

This study uses panel data of 11 provinces and cities in the Yangtze River Economic Belt from 2008 to 2016, while a panel threshold model is constructed to explore the non-linear relationship between FDI and carbon emissions intensity from the perspective of marketization level. The main conclusions are as follows:

First, from the perspective of marketization level, a significant double threshold effect exists between foreign participation and carbon emissions intensity, with threshold values of 4.4701 and 9.2516, respectively. Second, as the marketization level increases, the technology spillover effect of FDI increases, and the stimulation effect of foreign participation on carbon intensity decreases significantly, but it does not inhibit carbon intensity, indicating that the overall benefits brought by FDI technology spillovers are still insufficient to offset pollution caused by foreign investment. Third, from 2008 to 2011, with the exception of Guizhou Province, the remaining 10 provinces and cities had marketization levels between the first and second thresholds, while no province or city crossed the second threshold. In 2012–2016, the marketization levels of Jiangsu and Zhejiang provinces were the first to cross the second threshold, while Guizhou Province gradually crossed the first threshold and entered the ranks between the first and second thresholds. In 2016, Chongqing became the only region in the central and western regions that successfully crossed the second threshold. Overall, the central and western regions of the Yangtze River Economic Belt are still firmly stuck between the first and second thresholds due to the relatively weak economic foundation and the relatively slow marketization process. Fourth, other control variables such as energy intensity, energy structure, industrial structure, and urbanization increase carbon intensity to varying degrees, while openness to the outside world and technological innovation inhibit carbon intensity.

### Policy Implications

In view of the research conclusions, the following policy suggestions are put forward from three dimensions, namely the strategies for introducing foreign investment, marketization construction, and environmental regulation, in the hope of realizing the inhibitory effect of FDI on carbon emissions intensity and promote the development of a green economy.

In terms of introducing foreign investment, on the one hand, the opening up must be continuously deepened in accordance with “negative list” management. On the other hand, it is necessary to make plans for introducing investment based on the actual situations of industrial development in the region, to avoid blindly introducing capital with the aim of short-term political achievements. The focus should be on introducing foreign investments that are technology-based, low-carbon and green, and have strong industrial linkage, so that the investment can be embedded in the local industrial chain and promote more technology spillovers. At the same time, to raise the effectiveness of FDI technology spillovers, there should be focus on cultivating the innovative and learning ability of local enterprises, strengthening the cooperation with foreign enterprises, and shortening the learning cycles of technology.

In terms of marketization construction, since a suitable degree of economic agglomeration is more conducive to releasing the effect of FDI on green development, market rules should be followed at this stage, while cities in the eastern region should be encouraged to implement a deeply advancing development model, with a compact and intensive nature. It is not appropriate to use excessive administrative measures to restrict the concentration of population in these regions. While seeking to maximize the benefits of economic agglomeration in the eastern region, the bearing capacity of the region in terms of economy, infrastructure, as well as resources and environment should also be considered, to avoid negative impacts caused by excessive agglomeration. It is necessary to induce the gradual flow of FDI, industries, and population toward the central and western regions, so as to form a more rationally distributed hierarchical structure of cities, and improve the overall efficiency of the green economy, and achieve coordinated development between regions. In addition, governments should strengthen the construction of the legal system, improve the protection of intellectual property rights for low-carbon technologies, and encourage enterprises to increase R&D investment (Tan et al., 2019).

In terms of environmental regulations, reasonable environmental regulations and effective implementation will keep foreign investment that produces high-pollution and high-emission away. A new assessment system should be introduced to include green development factors in the assessment scope of local governments, to guarantee effective implementation of environmental regulations and avoid a “race to the bottom.” Specifically, it is necessary to cancel the advantageous policies for FDI enterprises gradually, and implement reasonable guidance and supervision to the enterprises, so as to reduce the negative effect brought by FDI to the industrial structure. Governments should encourage and support the technology introduction and independent research that are conducive to cleaner production and resources-saving, to better utilize the technology spillover effect of FDI in energy-saving and environmental protection and strengthen the coordination and linkage of open development and green development.

## Data Availability Statement

The original contributions presented in the study are included in the article/supplementary material, further inquiries can be directed to the corresponding author/s.

## Author Contributions

All authors undertook research, writing, and review tasks throughout this study. All authors have read and agreed to the published version of the manuscript.

## Conflict of Interest

The authors declare that the research was conducted in the absence of any commercial or financial relationships that could be construed as a potential conflict of interest.

## Publisher’s Note

All claims expressed in this article are solely those of the authors and do not necessarily represent those of their affiliated organizations, or those of the publisher, the editors and the reviewers. Any product that may be evaluated in this article, or claim that may be made by its manufacturer, is not guaranteed or endorsed by the publisher.
